# Prompt engineering and diagnostic accuracy of multimodal large language models in thyroid fine-needle aspiration cytology

**DOI:** 10.6026/97320630021317

**Published:** 2025-06-30

**Authors:** Bibhas Saha Dala1, Kaushik Mukhopadhyay, Dwaipayan Roy, Souvik Bhattacharya, Indranil Chakrabarti, Santosh Kumar Mondal

**Affiliations:** 1 Department of Pathology, All India Institute of Medical Sciences (AIIMS), Kalyani, West Bengal, India; 2 Department of Pharmacology, All India Institute of Medical Sciences (AIIMS), Kalyani, West Bengal, India; 3 Department of Computational and Data Sciences, Indian Institute of Science Education and Research (IISER), Kolkata, West Bengal, India

**Keywords:** Fine-needle aspiration cytology, large language models, thyroid nodule, artificial intelligence, prompt engineering, diagnostic accuracy

## Abstract

Role of Large language models (LLMs) in fine-needle aspiration cytology (FNAC) image analysis remain uncertain. We evaluated two LLMs - Chat GPT-4o (OpenAI) and Claude 3.5 Sonnet (Anthropic) on 63 thyroid FNAC cases, each represented by eight microscopic images (Pap and MGG, 10x/40x), using generic and structured prompts. Structured prompts improved Bethesda concordance and near-match rates but inter-rater agreement remained poor (κ ≤ 0.09). Specificity reached 100% with structured prompts, but sensitivity dropped to ≤11.8% and misclassification persisted. LLMs show potential, but domain-specific training and validation are necessary for clinical use.

## Background:

Thyroid diseases are quite prevalent worldwide and often present as thyroid nodules [1, 2]. Over 95% of these nodules are benign; approximately 5% may harbour malignancy. No individual diagnostic modality, such as ultrasonography or scintigraphy, can definitively distinguish between benign and malignant thyroid lesions [3]. Fine-needle aspiration cytology (FNAC) has proven reliable, cost-effective and time-efficient for diagnosing and managing thyroid disorders, especially with ultrasound guidance. It reduces unnecessary thyroid surgeries and remains the most valuable preoperative diagnostic tool due to its safety and simplicity in detecting benign vs. malignant lesions [2, 3-4]. To standardise reporting and improve communication among clinicians, the 2023 Bethesda System for Reporting Thyroid Cytopathology (TBSRTC) was proposed, which offers a standardised, structured, category-based framework for reporting findings from thyroid fine-needle aspiration samples. It classifies thyroid FNAC samples into six categories: I - Non-diagnostic, II - Benign, III - Atypia of Undetermined Significance (AUS), IV - Follicular Neoplasm, V - Suspicious for Malignancy and VI - Malignant [5]. This system has become a cornerstone of thyroid cytopathology, guiding clinical decision-making based on cytological categories. As in many other domains, Artificial Intelligence (AI) has increasingly found applications in medicine over the past decade and pathology is no different. While it has not yet replaced the traditional microscope, advances in digital pathology have paved the way for AI to integrate into some regions of pathology practice [6, 7]. More recently, the emergence of generative large language models (LLMs), such as Chat GPT, Claude, *etc.*, has revolutionized several domains in healthcare. Originally developed for text-based tasks such as reasoning, answering questions and translation, these models have now expanded their capabilities to process images and produce descriptive and diagnostic outputs [8, 9]. While large language models offer promising potential in pathology image analysis, their inconsistent diagnostic accuracy, limited interpretability and a lack of true medical contextual understanding present significant challenges. The potential for these models to generate incorrect results or hallucinations necessitates careful consideration. Without rigorous validation and expert oversight, the integration of LLMs into clinical workflows could introduce risks to patient safety and the integrity of medical decision-making [10, 11]. LLMs have been scarcely explored for cytology image analysis in existing literature. Therefore, it is of interest to report that gap by evaluating the diagnostic performance of Chat GPT and Claude in thyroid FNAC, comparing their image-based interpretations to the consensus diagnoses provided by expert cytopathologists.

## Methodology:

## Image collection and organization:

Our study encompassed a total of 63 thyroid FNAC cases. For each case, expert cytopathologists meticulously selected eight representative digital JPEG images with varying staining and magnification scales, ensuring a comprehensive dataset. Specifically:

[1] Two images stained with Papanicolaou (Pap) stain at 10x magnification.

[2] Two images stained with Pap stain at 40x magnification.

[3] Two images stained with May-Grünwald-Giemsa (MGG) stain at 10x magnification.

[4] Two images stained with MGG stain at 40x magnification.

Were selected for the construction of the dataset and these images were saved in individual folders labelled numerically from 1 to 63, with each folder representing a single case. The images within each folder were consistently numbered from 1 to 8 to maintain image type and magnification order. Bethesda Category III (AUS) was excluded from this study due to its high interobserver variability, diagnostic ambiguity not suitable for AI interpretation and to maintain a focus on clinically actionable categories (benign vs malignant) for clearer assessment of AI model performance.

## AI-based analysis using LLMs:

Two state-of-the-art multimodal large language models were selected for evaluation in this study: Chat GPT, specifically the "Chat GPt-4o-latest" version from OpenAI and Claude, identified as 'claude-3-5-sonnet-20240620' from Anthropic. Both models were accessed on 9th January, 2025, using their respective APIs with vision capability enabled. A Python-based automation pipeline was developed to facilitate bulk submission of images to each model. For each LLM, the script uploaded all eight images simultaneously to the respective LLM, accompanied by the following initial diagnostic prompt:

"Analyse these thyroid FNAC images from a {age} year old {sex} patient. Provide a detailed analysis using EXACTLY these headers:

[1] Cellularity: (specify Low/Moderate/High)

[2] Cell patterns: (describe architectural arrangement in max 20 words)

[3] Nuclear features: (describe nuclear characteristics in max 20 words)

[4] Background: (describe background elements in max 20 words)

[5] Notable findings: (list significant observations in max 20 words)

[6] Final diagnosis: (provide specific diagnostic impression)

[7] Bethesda category: (specify category I-VI)

## Important:

[1] Use EXACTLY these headers

[2] Keep descriptions concise within word limits

[3] Ensure that Final Diagnosis and Bethesda Category are explicitly stated."

For this study and based on the specific large language models employed, we will refer to the OpenAI 'Chat GPt-4o-latest' system as OpenAI and the Anthropic 'claude-3-5-sonnet-20240620' system as Claude. This consistent nomenclature will be used throughout our analysis and discussion. The outputs (cytological description, Bethesda category and diagnosis) were collected for each case and saved in a structured. CSV file for further analysis.

## Prompt revision and re-evaluation:

A second round of analysis was conducted using revised prompts for both LLMs to evaluate the influence of prompt design on model performance. The revised prompt provided more specific guidance regarding diagnostic criteria and requested a clearer rationale behind the assigned category:

"You are a conservative pathology expert specialized in analysing thyroid FNAC (Fine Needle Aspiration Cytology) images.

Your approach is careful and methodical, following strict criteria for malignancy diagnosis. You understand that:

[1] Over diagnosis of malignancy can lead to unnecessary procedures

[2] Clearly, benign features should be acknowledged

[3] Suspicious features must meet multiple established criteria before suggesting malignancy

Focus on objective cytological features visible in the images and provide a structured, evidence-based analysis. Analyse these thyroid FNAC images from a {age} year old {sex} patient. Consider all findings carefully before making a diagnosis. Provide analysis using EXACTLY these headers:

Cellularity: (specify Low/Moderate/High)

Cell Patterns: (describe architectural arrangement in max 20 words, focus on clear, definitive patterns)

Nuclear Features: (describe nuclear characteristics in max 20 words, note both benign and atypical features if present)

Background: (describe background elements in max 20 words)

Notable Findings: (list significant observations in max 20 words, including any benign characteristics)

Final Diagnosis: (provide definitive diagnostic impression, acknowledging if features are borderline)

Bethesda Category: (specify category I-VI)

## Important:

[1] Use EXACTLY these headers

[2] Keep descriptions concise within word limits

[3] Ensure Final Diagnosis and Bethesda Category are explicitly stated

[4] List both benign and atypical features when present"

Keeping the nomenclature consistent, we will refer to the systems with revised prompts as OpenAI Revised and Claude Revised. This will ensure clarity and consistency when discussing the impact of prompt modifications on the performance of the models. Outputs from this revised analysis were similarly saved in a separate. csv file for comparison.

## Expert comparison and evaluation:

The results from both original and revised prompts were compared to the gold-standard Bethesda categories and diagnoses assigned by expert cytopathologist through blinded consensus review. Additionally, for each AI-generated report, expert pathologists provided qualitative feedback categorized as: Accurate, Acceptable and Not Acceptable. These assessments were used to evaluate each model's clinical utility and reliability. Diagnostic concordance, exact match rates, ± 1 category deviation, major mismatches (≥2 category difference) and inter-rather agreement (Cohen's kappa) were calculated. Confusion matrices and binary classification metrics (benign vs malignant) were also derived. All data processing, prompt engineering, image management and result evaluation were conducted using Python (v3.11) and relevant libraries such as Pandas, OpenAI and Anthropic.

## Results and Discussion:

Summary of demographic and diagnostic data for all cases include in the study. Age is expressed as mean ± standard deviation. The Bethesda category distribution is based on cytopathologist evaluation. The five most common final cytology diagnoses are also listed. Comparison of the degree of agreement between models-generate Bethesda categories and the ground truth from pathologists. Results are presented as exact matches, near matches (±1 category deviation) and major discrepancies (≥2 category difference). Inter-rater reliability is reported using Cohen's kappa coefficient. Each panel presents a confusion matrix for one AI model version, showing the agreement between predicted and actual Bethesda category (I-VI). Darker cells indicate higher frequency. Diagonal elements represent correct predictions. Axis labels denote pathologist-assigned (true) and AI-generated (predicted) categories. Side-by-side binary confusion matrices for each AI model compare predictions (Benign vs Malignant) against the pathologist reference. Metrics displayed below each matrix include accuracy, sensitivity, specificity, PPV and NPV. Subpanels are annotated (a-d) and axis order is consistent across all plots. Bar plot showing the frequency and percentage of pathologist feedback per model, categorized as Accurate, Acceptable, or Not Acceptable. Each bar represents the number of cases, with percentage labels above. This visualization reflects subjective expert appraisal of AI-generated outputs.

## Baseline characteristics:

We analysed 63 thyroid FNAC cases from patients with a mean age of 41.4 ± 11.7 years, predominantly female (n=47, 74.6%). Pathologist-assigned Bethesda categories included category I (n=4, 6.3%), category II (n=37, 58.7%), category IV (n=5, 7.9%), category V (n=7, 11.1%) and category VI (n=10, 15.9%), with no category III cases. The most frequent diagnoses were colloid goitre (n=23, 36.5%), lymphocytic thyroiditis (n=14, 22.2%) and papillary carcinoma (n=8, 12.7%) (Table 1 - see PDF)

## AI performance:

Variable concordance between AI-generated and pathologist-assigned Bethesda categories is demonstrated in (Table 2 - see PDF). Open AI Revised achieved the highest exact category match rate (28.6%, n=18), followed by Claude Revised (22.2%, n=14). When including adjacent category assignments (±1 category), Claude Revised performed best (74.6%, n=47), with OpenAI Revised second (61.9%, n=39). Major discrepancies (≥2 category difference) were most common with the original Claude model (61.9%, n=39) and least frequent with Claude Revised (25.4%, n=16). Cohen's kappa values were consistently poor across all models (range: 0.03-0.09), indicating minimal agreement beyond chance. The confusion matrices (Figure 1 - see PDF) reveal that all AI models disproportionately assigned Bethesda category II and over-predicted categories IV and V. The revised models showed improved alignment with pathologist classifications compared to their original counterparts.

## Binary classification performance:

For the clinically relevant benign versus malignant distinction (Figure 2 - see PDF), Claude Revised achieved the highest accuracy (76.2%), closely followed by OpenAI Revised (74.6%). The original models demonstrated substantially higher sensitivity for malignancy (both 70.6%) but suffered from lower specificity (58.7% for OpenAI, 41.3% for Claude). Conversely, both revised models achieved 100% specificity but at the cost of markedly reduced sensitivity (5.9% and 11.8% for OpenAI Revised and Claude Revised, respectively). This pattern manifested in the confusion matrices, where revised models classified nearly all cases as benign, correctly identifying benign cases but missing most malignancies. The original models identified more true malignancies but generated numerous false positives.

## Qualitative assessment by pathologists:

Pathologist feedback (Figure 3 - see PDF) revealed that Claude Revised received the highest combined proportion of "Accurate" and "Acceptable" ratings (74.6%), followed by OpenAI Revised (55.6%). The original models performed less favourably, with combined ratings of 38.1% for OpenAI and 34.9% for Claude. The proportion of "Not Acceptable" diagnoses-those considered clinically misleading or inappropriate-was lowest for Claude Revised (25.4%) and highest for the original Claude model (65.1%). This qualitative assessment aligns with the quantitative metrics, confirming that revised models produced more clinically appropriate interpretations. The data present a complex picture of AI performance in thyroid cytopathology. While revised models demonstrated marked improvement over their original versions, particularly for benign case identification, they still exhibited significant limitations. The poor kappa values across all models, combined with low sensitivity for malignancy in the revised versions and low specificity in the original versions, indicate that none of these AI systems can be reliably used in clinical practice. The best-performing model, Claude Revised, still misclassified 25.4% of cases in a manner deemed clinically unacceptable by pathologists. These findings highlight the current limitations of AI in thyroid cytopathologic diagnosis and underscore the continued necessity of human expert assessment for accurate FNAC interpretation. This study represents one of the earliest attempts to evaluate large language models (LLMs), specifically Chat GPT (GPT-4o Vision) and Claude 3.5 Sonnet, in the interpretation of thyroid fine-needle aspiration cytology (FNAC) images using the 2023 Bethesda System. While LLMs have gained recognition in medical imaging domains such as dermatology, radiology and histopathology [9, 12] their application in cytopathology remains largely unexplored. Our study thus provides important insights into the potential and limitations of LLMs in this domain.

LLMs have shown significant promise in the medical field, but their diagnostic reliability remains closely tied to prompt engineering, which directly influences the clarity and relevance of model outputs. Studies by Russe *et al.* and Nguyen *et al.* have emphasized that well-structured prompts significantly enhance LLM performance, while also highlighting the inconsistent and sometimes hallucinatory outputs produced by models like Chat GPT-4 when analysing public images [13, 14]. Similarly, Wang *et al.* reported that the reliability of LLM outputs across different prompts varied widely, with Kappa Coefficient ranging from -0.002 to 0.984, indicating instability in consistency and agreement depending on prompt formulation and context [15]. Our study reinforces these findings in the domain of cytopathology, showing that the transition from original to revised prompts led to measurable improvements in diagnostic performance for both Chat GPT and Claude. For Chat GPT, exact match rates improved from 15.9% to 28.6%, ±1 category deviations from 28.6% to 33.3% and major discrepancies decreased from 55.6% to 38.1%, with a modest rise in Cohen's kappa from 0.03 to 0.05. Claude demonstrated even greater gains, with exact matches increasing from 12.7% to 22.2%, near matches from 25.4% to 52.4%, major discrepancies dropping from 61.9% to 25.4% and kappa improving from 0.04 to 0.09. These results underscore the impact of prompt refinement on model performance in complex image-based diagnostic tasks like thyroid FNAC. However, despite these improvements, the overall diagnostic concordance and inter-rater reliability remain below clinically acceptable standards when compared to expert cytopathologist assessments, highlighting the need for further development and validation before LLMs can be reliably integrated into routine cytological diagnostics.

Building on the growing body of literature, our study contributes to the understanding of large language models in the context of image-based cytopathologic diagnostics, specifically thyroid FNAC. Agbareia *et al.* demonstrated that while LLMs outperformed physicians in text-only diagnostic scenarios, current multimodal LLMs are not yet fully optimized for visual interpretation tasks [16]. Yang *et al.* emphasized the transformative potential of LLMs across various medical domains, including pathology; however, they also pointed to significant challenges such as algorithmic bias, hallucinations and the critical need for rigorous clinical validation and ethical oversight [17]. Suh *et al.* echoed these concerns in a radiologic context, showing that while GPT-4o performed better than some less-experienced human readers, its diagnostic accuracy remained inferior to that of experienced radiologists and was not significantly improved by adding image inputs. They also highlighted that text input length significantly influenced LLM accuracy, with longer cases yielding better results [18]. Our findings align with these observations. While the binary classification analysis showed that original prompts given to the models (Chat GPT and Claude) had high sensitivity (70.6%) but low specificity (58.7% and 41.3%, respectively), the revised prompts reversed this pattern, demonstrating perfect specificity (100%) but low sensitivity (5.9% and 11.8%). This reflects the broader challenge of LLMs balancing diagnostic conservatism and overdiagnosis, likely influenced by prompt structure and inherent model biases. Expert feedback further supported these quantitative results, identifying Claude Revised as the most clinically acceptable model (74.6% "Accurate" or "Acceptable" responses), though it still misclassified 25.4% of cases in a clinically unacceptable manner. Even with the exclusion of Bethesda Category III (AUS/FLUS) to avoid interpretative ambiguity, the LLMs struggled to correctly classify well-defined cases. This highlights the inherent complexity of cytology, which depends on nuanced morphological patterns and contextual integration - skills that LLMs currently lack. As echoed across recent studies, general-purpose LLMs trained on broad datasets fall short when applied to domain-specific medical tasks without further tuning.

## Conclusion:

Our study reinforces existing concerns about the readiness of LLMs for clinical cytology use. While there is clear potential, particularly with structured prompt engineering and the promise of multimodal capabilities, current models are not yet accurate or reliable enough to replace expert cytopathologist judgment. Advancing LLM performance in this domain will require domain-specific model training, multimodal learning optimization and strong interdisciplinary collaboration to ensure safe and clinically effective integration into diagnostic workflows.
